# Assessment of Primary Stability and Micromotion of Different Fixation Techniques for Scapular Spine Bone Blocks for the Reconstruction of Critical Bone Loss of the Anterior Glenoid—A Biomechanical Study

**DOI:** 10.3390/life15040658

**Published:** 2025-04-16

**Authors:** Anton Brehmer, Yasmin Youssef, Martin Heilemann, Toni Wendler, Jean-Pierre Fischer, Stefan Schleifenbaum, Pierre Hepp, Jan Theopold

**Affiliations:** 1 Department of Orthopedic, Trauma, and Plastic Surgery, University of Leipzig, Liebigstraße 20, 04103 Leipzig, Germany; anton@brehmermail.de (A.B.); martin.heilemann@medizin.uni-leipzig.de (M.H.); toni.wendler@medizin.uni-leipzig.de (T.W.); jean-pierre.fischer@medizin.uni-leipzig.de (J.-P.F.); stefan.schleifenbaum@medizin.uni-leipzig.de (S.S.); pierre.hepp@medizin.uni-leipzig.de (P.H.);; 2 ZESBO—Center for Research on Musculoskeletal Systems, Semmelweisstraße 14, 04103 Leipzig, Germany

**Keywords:** shoulder instability, scapular spine, bone block augmentation, cerclage technique, Latarjet procedure, micromotion, primary stability

## Abstract

Anteroinferior shoulder dislocations require surgical intervention when related to critical glenoid bone loss. Scapular spine bone blocks have emerged as a promising alternative to traditional bone augmentation techniques. However, limited data exist on their biomechanical stability when using different suture-based fixation techniques. This study aimed to evaluate primary stability and micromotion after glenoid augmentation using a scapular spine bone block. A total of 31 fresh-frozen human shoulder specimens underwent bone block augmentation. The specimens were randomized into three groups: double-screw fixation (DSF), single-suture bone block cerclage (SSBBC), and double-suture bone block cerclage (DSBBC). Biomechanical testing was conducted using cyclic loading (5000 cycles at 1 Hz) and micromotion was analyzed using an optical 3D measurement system. Statistical analysis showed that medial irreversible displacement was significantly greater in the SSBBC group compared to DSF (*p* = 0.0386), and no significant differences were found in anterior or inferior irreversible displacements. A significant difference was noted in posterior reversible displacement (*p* = 0.0035), while no differences were found in inferior or medial reversible displacements. Between DSF and DSBBC, no significant differences were found in irreversible or reversible displacements in any direction. DSBBC provided stability comparable to DSF while offering a viable metal-free alternative. In contrast, SSBBC displayed inferior biomechanical properties, raising concerns about its clinical reliability.

## 1. Introduction

The most common joint dislocation in humans is that of the shoulder [1]. Up to 95% of these are anteroinferior [2,3,4]. In the current literature, both non-surgical and surgical treatment options have been described for anteroinferior shoulder dislocations [5].

In recurrent shoulder dislocations or patients with a significant glenoid defect, conservative treatment [6] or treatment of soft tissue damage alone [7] is often not sufficient, and both are associated with high recurrence rates [8,9,10]. The cut-off values for critical glenoid bone loss, which clearly necessitates bone block augmentation, have been progressively lowered in the past years. Bigliani et al. [11] initially defined a 25% bone defect as critical, while the current cut-off value for critical glenoid bone loss is set at around 15% [12,13]. However, the cut-off values and measurement methods remain a topic of ongoing debate [14]. For clinical decision making, the literature clearly recommends a bone block procedure for bone defects greater than 20%. And, for bone defects between 15 and 20%, soft tissue repair can be considered [15].

Frequently used bone augmentation procedures are the Latarjet procedure, iliac crest autograft, and distal tibia allograft [16,17,18,19]. These are, however, all associated with significant complications including nerve damage, vascular injury, infection, intraoperative fractures, and persistent donor site pain [20,21,22]. Autologous scapular spine bone blocks have recently been suggested to address these associated challenges and complications [23]. It has been shown that scapular spine bone blocks are comparable to gold-standard procedures (Latarjet, iliac crest autograft). Rohmann et al. [24] demonstrated that scapular spine bone block dimensions are comparable to those of bone blocks of the coracoid or iliac crest. In a controlled laboratory study involving 20 human cadavers, Kuan et al. [25] demonstrated that a scapular spine graft exhibited greater stiffness and no inferiority in failure loads compared to a coracoid graft. Biomechanically, Youssef et al. [26] found comparable primary stability as well as comparable micromotion in screw-fixed scapular spine blocks and coracoid blocks.

In screw-fixed bone augmentations, screws may, however, loosen, migrate, or even break, potentially leading to complications such as soft tissue irritation or the need for hardware removal [27,28]. To address these issues, metal-free procedures, including suture-based techniques, have been developed. These approaches may offer advantages in terms of improved postoperative healing, reduced foreign body reactions, and a lower risk of implant-associated complications [29,30]. Biomechanical tests and clinical comparisons have not demonstrated any inferiority of metal-free techniques [31]. Some studies even suggest that suture-based systems are biomechanically superior to screw fixation [32]. There, however, remains a lack of studies examining micromotion and primary stability and therefore suitability in suture-based systems for the reconstruction of the glenoid with a spina scapula bone block.

The purpose of this biomechanical study was to determine primary stability and micromotion at the glenoid–bone block interface for three different fixation techniques, double-screw fixation (DSF), single-suture bone block cerclage (SSBBC), and double-suture bone block cerclage (DSBBC), when using a scapular spine bone block in critical glenoid bone loss.

## 2. Materials and Methods

A total of 31 fresh-frozen human shoulders were available for testing: 16 right and 15 left shoulders, with 14 paired and three unpaired shoulders. Eleven of the shoulders were from female donors and twenty were from male donors. The median age of the donors was 82.1 years (SD: 11.4 years). The shoulders were obtained from the Institute of Anatomy, Leipzig, Germany.

### 2.1. Ethical Implications

Being part of the body donor program regulated by the Saxonian Death and Funeral Act of 1994 (Section 3, paragraph 18, item 8), institutional approval for the use of the postmortem tissues of human body donors was obtained. In addition, an institutional IRB approval in favor of the use of body donor material was obtained by the Ethics Committee of the Medical Faculty of the University of Leipzig (129/21-ek).

### 2.2. Specimen Preparation

The shoulders were stored at −80 °C and thawed at 3 °C for 24 h prior to biomechanical testing. Clinical data on each of the doners were obtained from the Institute of Anatomy. None of the doners had clinically diagnosed osteoporosis or osteoarthritis. Before conducting the tests, all surrounding soft tissue was removed and the shoulders were examined. All specimen with fractures or visible signs of degenerative changes were excluded from this study.

The middle section of the scapular spine was harvested from each cadaver to create a bone block for glenoid augmentation [24]. An artificial 20% bone defect was created at the glenoid to simulate bone loss. A 20% bone defect was chosen according to the current literature. Bone blocks are only clearly recommended for defects exceeding 20% [15]. All bone block harvesting and glenoid defect placement were performed by an experienced senior surgeon specializing in shoulder surgery (J.T.).

The shoulders were randomized and assigned to the following 3 test groups:

- DSF group: Scapular spine bone block augmentation using double-screw fixation (*n* = 11)
- SSBBC group: Scapular spine bone block augmentation using single-suture bone block cerclage (*n* = 10)
- DSBBC group: Scapular spine bone block augmentation using double-suture bone block cerclage (*n* = 10)

### 2.3. Glenoid Augmentation Procedure

The surgeries were performed by the same senior surgeon as who performed the specimen preparation (J.T.). Two holes were drilled in the anterior glenoid rim and two holes were drilled in the bone block, 1 cm apart, on all shoulders. The bone blocks were fixed either with screws or one of the two suture-based systems according to randomization.

#### 2.3.1. Double-Screw Fixation

**Figure 1:**
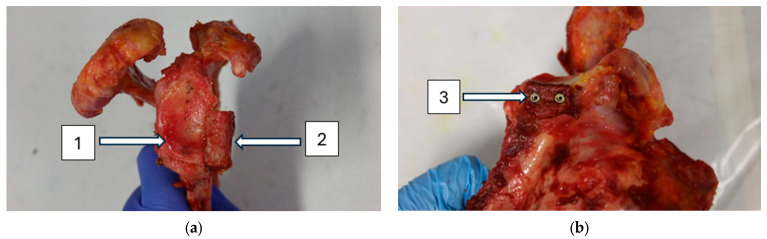
Double-screw fixation using a scapular spine bone block (**a**,**b**): (1) glenoid, (2) scapular spine bone block, (3) two cannulated compression screws.

In the DSF group, the bone block was affixed to the glenoid using two cannulated compression screws (DePuy Synthes, Zuchwil, Switzerland) as shown in Figure 1. One screw was inserted through the more cranial holes of the bone block and glenoid, and the other screw was inserted through the more caudal holes of the bone block and glenoid.

#### 2.3.2. Single-Suture Bone Block Cerclage

**Figure 2:**
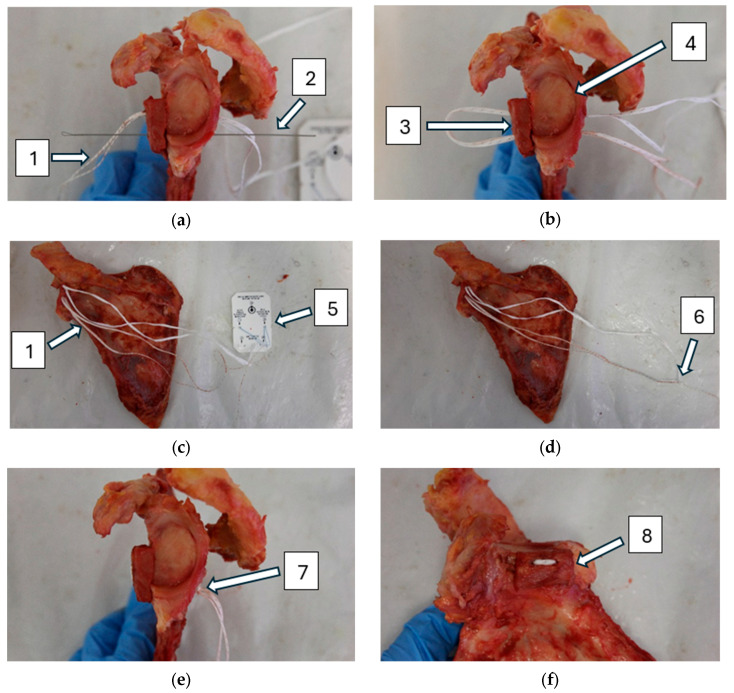
Single-suture cerclage technique using scapular spine bone block (**a**–**f**): (1) TigerTape, (2) suture shuttle, (3) scapular spine bone block, (4) glenoid, (5) loader, (6) knot, (7) knot secured with additional half hitch, (8) bone block affixed to glenoid.

In the SSBC group, the scapular spine bone block was attached to the glenoid using TigerTape (Arthrex, Naples, FL, USA) as illustrated in Figure 2. The TigerTape was passed posterior to anterior through the cranial hole, then through the bone block, and back anterior to posterior through the caudal hole (Figure 2a,b). The end of the suture was inserted into the knot (at the other end of the suture) using a loader (Figure 2c). By pulling the sutures alternately, the knot was pushed forward to the back of the glenoid (Figure 2d). Then, the knot was tightened slightly using a tensioner. A half hitch was used to secure the knot and was also tightened using a tensioner. Finally, 3 more alternating half-hitches were applied to the knot and the excess end was cut off (Figure 2e,f).

#### 2.3.3. Double-Suture Bone Block Cerclage

**Figure 3:**
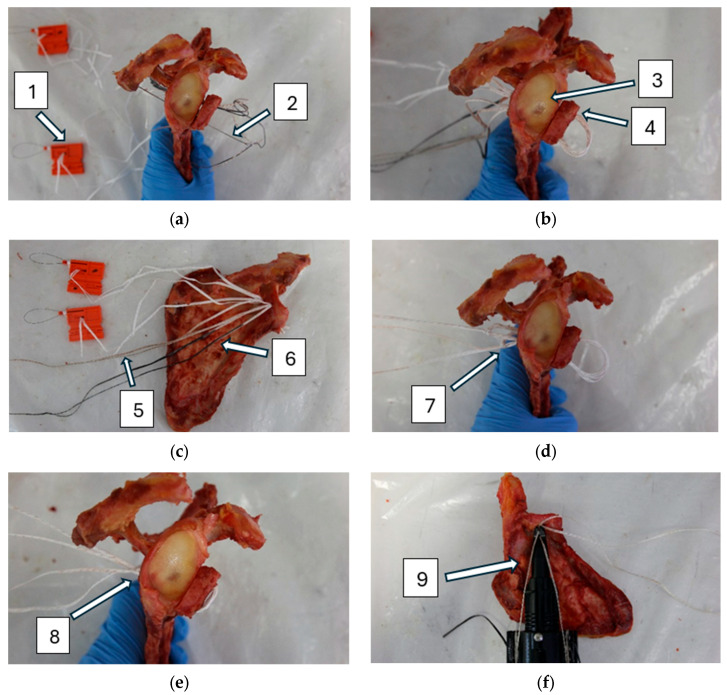
Double-suture cerclage technique using scapular spine bone block (**a**–**f**): (1) loader, (2) suture shuttle, (3) glenoid, (4) scapular spine bone block, (5) TigerTape, (6) FiberTape (marked in black), (7) knots, (8) knots secured with additional half hitches, (9) tensioner.

In the DSBBC group, the bone block was affixed to the glenoid using blue/white TigerTape (Arthrex, Naples, FL, USA) and black FiberTape (Arthrex, Naples, FL, USA). The surgical procedure is shown in Figure 3. The black FiberTape and blue/white TigerTape were passed posterior to anterior through the cranial hole, then through the bone block, and back anterior to posterior through the caudal hole (Figure 3a,b). The end of the TigerTape suture (blue/white) was inserted into the knot of the FiberTape suture (marked with a black dot for identification) using a loader. The end of the FiberTape suture (black) was inserted into the knot in the TigerTape suture (un-marked knot) using a loader (Figure 3c). By pulling the sutures alternately (behind the knots), the two knots were advanced to the back of the glenoid (Figure 3d,e). Then, the knots were tightened slightly using a tensioner (Figure 3f). Half hitches were used to secure both knots and these were also tightened using a tensioner. Finally, 3 more alternating half-hitches were applied to the knots and the excess ends were cut off.

### 2.4. Biomechanical Testing

The shoulders were embedded in a quick-setting resin (polyol + isocyanate + aluminum hydroxide). This embedding material was chosen for its rapid curing properties, ensuring the stable fixation of the specimens during testing while minimizing potential thermal effects on the bone structure. Unlike other resins that generate significant exothermic reactions during polymerization, this specific formulation releases only minimal heat. Excessive heat exposure could potentially alter the mechanical properties of bone by affecting collagen integrity or inducing microstructural changes. However, due to the low thermal output of the selected resin, any impact on the biomechanical properties of the specimens could be considered negligible, preserving the validity of the experimental results. The glenoid was placed perpendicular to the axis of force application of the test device in the embedding tray. To record, the micromotion markers were affixed to the scapular spine bone block and to the glenoid using thin K-wires.

**Figure 4:**
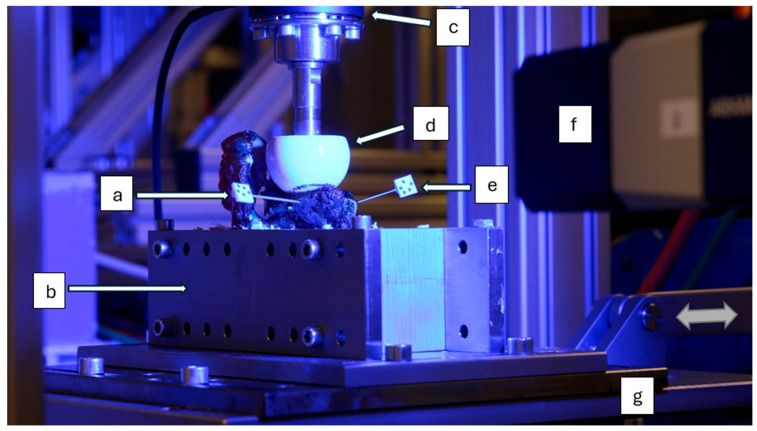
Biomechanical test setup: a: marker affixed to glenoid; b: embedding tray; c: force–torque sensor; d: ceramic head component; e: marker affixed to bone block; f: optical 3D measuring system; g: dynamic platform.

Prepared in this way, everything was clamped in the test device, based on the rocking-horse method described previously by Youssef et al. [26] (Figure 4). The test device consisted of a ceramic head component with a 44 mm head diameter that exerted a constant uniaxial force of 170 N on the scapula. The ceramic head component was moved across the glenoid surface horizontally. Two end-positions were defined for one movement cycle, which were the center of the initial glenoid and, secondly, the center of the bone block. The force of 170 N was chosen, as Bergman et al. have shown that glenohumeral contact forces during glenohumeral flexion and abduction can be up to 170 N even before the perceptible motion of the joint [33]. In the clinical setting, patients that have received a bone block will be immobilized in a shoulder sling for 3 to 6 weeks [34], so that the applied forces simulate the possible post-operative forces within the shoulder joint during early rehabilitation. During axial loading, a servo drive (BMH0703T01A2A and LXM32MD18M2, Schneider Electric SE, Rueil-Malmaison, France) executed the anteroposterior movement of the scapula via an eccentric disk. This movement resulted in an alternating loading of the glenoid and the bone block. In total, the test of an augmented glenoid consisted of 5000 cycles with a frequency of 1 Hz.

During testing, the movements of the markers were recorded by an optical 3D measuring system (ZEISS ARAMIS 3D Camera, Carl Zeiss GOM Metrology GmbH, Braunschweig, Germany). The optical system recorded the movements at a frame rate of 25 Hz at specific points in time during the test run (cycles 5, 25, 45, 65, 100, 250, 500, 1000, 2500, and 4995), with four cycles before and after being recorded to calculate an average value.

Before testing, one reference frame was recorded, which represented the initial situation including a reference object with particularly placed tracking points for the definition of the coordinate system. This ensured that the *z*-axis always corresponded to the load axis and the *x*-axis always corresponded to the anteroposterior movement of the scapula.

The resulting data were evaluated using the software GOM Correlate Pro 2022(Carl Zeiss GOM Metrology GmbH, Braunschweig, Germany) and postprocessed with MATLAB R2019a (The MathWorks Inc., Natick, MA, USA). GOM Correlate Pro evaluation included the continuous measurement of a 6-degree-of-freedom-distance between two local coordinate systems. These were attached to the respective markers with their initial origin inside the glenoid–block interface and their initial coordinate axis equal to the coordinate systems defined in the reference frame. During postprocessing, the local minima and maxima of the measured distance were extracted using a low-pass filter to reduce the data to the two extremes of the head component position. The resulting micromotion data were divided into irreversible displacement and reversible displacement. Irreversible displacement referred to the translation of the bone block relative to the initial position, while reversible displacement referred to the spatial oscillation of the block caused by cyclic loading. Spatial oscillation is defined as small, repetitive movements within three-dimensional space that do not result in permanent positional change. Irreversible displacements are of clinical significance because they reflect the loosening of the block. The excessive loosening of the block may result in re-dislocation. Reversible displacements are of clinical significance because they represent interfragmentary micromovements that influence bone healing. Movement was recorded in three axes, as seen from the anatomical glenohumeral joint: x = +posterior/−anterior; y = +superior/−inferior; and z = +lateral/−medial.

### 2.5. Statistical Analysis

Statistical analysis and data processing were carried out using GraphPad Prism 10 (Graphpad Software Inc., San Diego, CA, USA). To assess the normality of the data, a Q-Q plot was used for visual inspection, while a Shapiro–Wilk test was conducted for quantitative evaluation. The normal distribution of the data was thus confirmed. A two-tailed unpaired *t*-test was performed in addition to the calculation of means. The two suture cerclage techniques were compared with the reference group (DSF). The significance level was set at *p* < 0.05.

## 3. Results

For all fixation techniques, the greatest proportion of displacement occurred within the first 10 cycles. The dominant direction of displacement was medial displacement, both for the suture-based bone block cerclages (DSBBC and SSBBC) and for screw fixation (DSF).

In the DSF group, three shoulders failed: two due to glenoid fractures during cyclic loading (one after the 1st cycle and the other after the 250th cycle) and one shoulder as it touched the edge of the embedding tray during the tests. In the SSBBC group, four shoulders failed, and one was excluded. Two shoulders touched the edge of the embedding tray (one after between 1000 and 2500 cycles and the other within the first 10 cycles) and one shoulder failed after 4995 cycles due to marker dislocation, while another failed due to a glenoid fracture after between 250 and 500 cycles. One shoulder was excluded because the suture cut through the bone block during preparation. In the DSBBC group, two shoulders failed due to glenoid fractures (one after between 2500 and 5000 cycles and the other within the first 10 cycles).

### 3.1. DSF vs. SSBBC

The medial irreversible displacement was significantly greater in the SSBBC group than in the DSF group (*p* = 0.0386). However, no significant difference was found in anterior (*p* = 0.2073) or inferior (*p* = 0.3561) irreversible displacements. A statistically significant difference was identified in the posterior reversible displacement (*p* = 0.0035), which contrasted with the anterior displacements of the other two surgical techniques. No significant difference was found in inferior (*p* = 0.3706) or medial (*p* = 0.7267) reversible displacements between the DSF and SSBBC groups.

### 3.2. DSF vs. DSBBC

For irreversible displacements, no significant difference was observed for any of the directions (anterior: *p* = 0.9748; inferior: *p* = 0.4585; medial: *p* = 0.5990) between the DSF and DSBBC groups. There was also no significant difference for reversible displacements in the anterior (*p* = 0.9567), inferior (*p* = 0.6100), or medial (*p* = 0.5727) direction.

**Figure 5:**
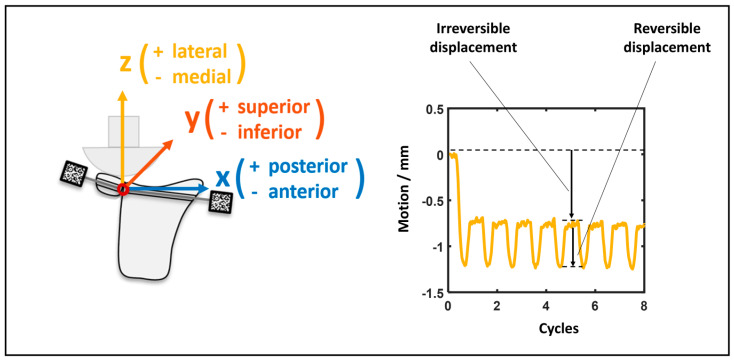
Graphical representation of reversible and irreversible displacements.

**Figure 6:**
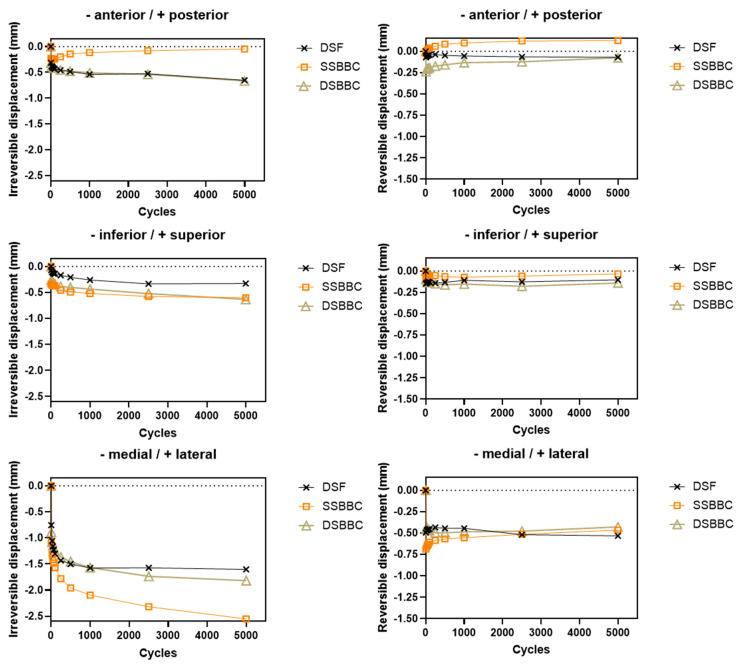
Mean irreversible and reversible displacements of respective groups (DSF, SSBBC, DSBBC) over 5000 cycles.

Table 1 presents the mean values and standard deviations for the irreversible and reversible displacements of the three groups (DSF, DSBBC, and SSBBC) after complete testing. Figure 5 is a schematic illustration of the reversible and irreversible displacements. Figure 6 shows the mean irreversible and reversible displacements of the respective groups over 5000 cycles.

## 4. Discussion

This study is particularly relevant as the gold-standard procedure for glenoid augmentation in critical bone defects, the Latarjet procedure, provides good primary stability but is also associated with significant complications, including screw migration, loosening, or even breakage. To address these issues, metal-free, suture-based fixation techniques have been developed in recent years and alternative bone graft harvesting sites have been introduced to reduce the risk of complications. Thus far, no study has investigated the biomechanical properties of metal-free fixation techniques in combination with a scapular spine bone block. The aim of this biomechanical study was to determine primary stability and micromotion at the interface of the glenoid and bone block for three different fixation techniques. The double-suture cerclage technique was shown to be comparable to double-screw fixation in terms of primary stability and micromotion. It was also found that single-suture bone block cerclage showed significantly higher micromotion differences compared to double-screw fixation.

Critical bone loss at the anterior glenoid is a major risk factor for recurrent anterior shoulder dislocation. Bone augmentation is recommended for bone loss greater than 15%. Scapular spine blocks have been described in the literature for glenoid augmentation and have already been biomechanically tested [25,26]. This study provides important insights into the biomechanical performance of scapular spine bone block fixation techniques for glenoid augmentation, comparing DSF, SSBBC, and DSBBC.

For all fixation techniques, the greatest proportion of irreversible displacement occurred within the first 10 cycles, emphasizing the importance of stability during the initial phase of fixation. The dominant direction of displacement was medial, which was consistent across all groups and refers to anatomical orientation based on the standard position of the shoulder in the human body. This observation is particularly relevant as medial displacement could compromise the integration of the bone block and the overall success of the procedure. Besides the risk of an increased rate of reluxation in the case of excessive medial displacement, Martins et al. [35] have shown that the incongruity caused by an excessive medial or lateral displacement can lead to an increase in peak contact pressures, which is associated with a higher risk of developing osteoarthritis.

Regarding the failure rate, glenoid fractures in which the bone block itself was not fractured were most likely due to poor bone quality. Even though each shoulder was examined for degenerative changes prior to testing, osteoporotic changes in the samples cannot be clearly ruled out. The other two failure patterns, contact with the edge of the embedding tray and the rotation of the markers, may be attributed to significant displacement between the bone block and the glenoid. While DSF exhibited three failures, primarily due to glenoid fractures, the SSBBC technique encountered the highest failure rate. The lowest failure rate was in the DSBBC group, with only two failures, both of which were due to a glenoid fracture. The higher failure rate of SSBBC compared to DSF and especially to DSBBC were most likely due to a greater level of micromotion, indicating reduced primary stability.

When comparing the DSF and SSBBC groups, no significant difference was found regarding anterior or inferior irreversible displacement. In contrast, irreversible displacement was significantly greater for SSBBC in the medial direction, which raises concerns about the suitability of single-suture cerclage in providing adequate fixation in vivo, particularly under repetitive loading conditions. A significant difference was also identified in posterior reversible displacement when comparing the SSBBC to DSF. This posterior movement—directed toward the glenoid—may have resulted from the lower initial stiffness and compressive stability of suture constructs compared to rigid screw fixation. However, the absolute magnitude of displacement remained minimal and within a clinically acceptable range, suggesting that this difference is unlikely to impact graft positioning or compromise clinical outcomes. However, no significant difference was observed for the inferior and medial reversible displacements.

When comparing the DSF and DSBBC groups, no significant difference was observed for any of the directions for irreversible and reversible displacements. The comparable performance of DSF and DSBBC in terms of irreversible displacements supports the potential of double-suture cerclage as a viable alternative to screw fixation, especially for patients where metal-free solutions are preferred. In contrast, the inferior performance of SSBBC, as indicated by greater displacements, suggests that single-suture cerclage may not provide sufficient primary stability for glenoid augmentation and is therefore not recommended to be used in clinical practice. The likely reason for this is that single-suture fixation distributes forces less effectively across the bone block, leading to increased micromotion at the interface. This instability can compromise initial bone healing, prolong recovery, and elevate the risk of fixation failure. Additionally, insufficient stabilization may result in the early loosening of the bone block, which could, in turn, increase the likelihood of re-dislocation and accelerate cartilage wear, ultimately contributing to the development of osteoarthritis. In contrast, double-suture cerclage provides enhanced fixation strength by more evenly distributing mechanical loads and reducing micromotion, thereby improving primary stability and potentially leading to better long-term clinical outcomes.

It has long been recognized that axial interfragmentary motion can promote bone healing [36]. For all three fixation techniques, the anterior and posterior reversible displacements were within the range described by Rechter et al. [37], which is thought to promote bone healing. In this study, the anterior and posterior reversible displacements reflected axial interfragmentary motion. Other studies also describe improved bone healing, increased osteogenic differentiation, or the avoidance of pseudarthrosis through small reversible displacements [38,39].

However, the literature confirms the encouraging results of this study in terms of the biomechanical properties of DSBBC. In a controlled laboratory study with a total of 60 human scapulae, Ritter et al. [40] described comparable primary stability between DSBBC and DSF. DSBBC is also being used successfully in practice and has been described in several technical notes [23,41,42]. Metal-free fixation techniques are alternatives to screw fixation. Initial studies on the clinical results of suture-based cerclages are promising. Hachem et al. [43] showed good functional outcomes, fewer implant-related complications, and improved radiographic assessment. However, it remains unclear whether bone healing rates differ between these approaches. There is limited but growing evidence that suture-based systems achieve comparable bone healing to screw fixation [44]. Suture-based techniques may offer biological advantages by reducing stress shielding and allowing for a more natural load distribution, potentially promoting better bone healing. Additionally, metal implants can induce inflammatory responses or fibrous encapsulation, whereas suture-based techniques might minimize these effects. However, there are limited comparative data on healing time and osseointegration between metal-free and screw-based fixation methods. Further research is needed to determine whether these differences translate into clinically relevant benefits.

### Limitations

The present study has some limitations linked to the nature of biomechanical tests. First, the mean age of the cadaveric specimens (82.1 years) does not reflect the typical patient population, which is generally younger [45,46]. The relatively large standard deviations observed in our results, particularly in relation to the mean values, highlight the inherent variability among the cadaveric specimens. Second, the removal of surrounding soft tissues may have altered the biomechanical properties of the specimens, limiting the replication of in vivo conditions. In a physiological setting, structures such as the joint capsule, ligaments, and periarticular muscles contribute significantly to joint stability by providing additional support and load distribution. The absence of these soft tissues in the experimental setup may have led to an overestimation of micromotion and displacement, as the bone block was solely reliant on its fixation construct. Additionally, muscle forces play a crucial role in stabilizing the shoulder joint, particularly during dynamic movements. Without their influence, the testing conditions may not fully reflect the mechanical environment experienced in vivo, potentially affecting the clinical relevance of the findings. Lastly, while this study used standardized cyclic loading protocols, the simulated conditions may not have fully captured the complexity of clinical scenarios, such as varying loading angles or patient-specific anatomical differences. However, despite these limitations, the setup provided a standardized and reproducible model for comparing different fixation techniques under controlled conditions. By isolating the mechanical properties of the fixation constructs, the study design allowed for a direct assessment of their relative stability, which is critical for evaluating their biomechanical performance in the early postoperative phase.

## 5. Conclusions

The double-suture bone block cerclage technique was comparable to double-screw fixation in terms of primary stability and micromotion. Single-suture bone block cerclage showed significantly increased micromotion differences compared to double-screw fixation. To confirm these findings and conclusively determine the most suitable augmentation procedure for each situation, further research with larger study populations, involving scapular spine blocks combined with suture fixation techniques, and long-term clinical outcomes is needed.

## Figures and Tables

**Table 1:** Mean values and standard deviations for the irreversible and reversible displacements of the three groups after 5000 cycles (DSF, DSBBC, and SSBBC).

Procedure	DSF	SSBBC	DSBBC
	Irreversible displacement/mm		
x	−0.65 ± 0.92	−0.05 ± 0.48	−0.67 ± 0.65
y	−0.33 ± 0.38	−0.60 ± 0.66	−0.63 ± 1.10
z	−1.60 ± 0.81	−2.55 ± 0.47 *	−1.82 ± 0.78
	Reversible displacement/mm		
x	−0.07 ± 0.10	0.13 ± 0.09 *	−0.08 ± 0.22
y	−0.10 ± 0.13	−0.03 ± 0.14	−0.14 ± 0.15
z	−0.53 ± 0.40	−0.47 ± 0.15	−0.43 ± 0.32

* Significant difference from comparison group (DSF group). Coordinate axes: x = +posterior/−anterior; y = +superior/−inferior; z = +lateral/−medial.

## Data Availability

The data that support the findings of this study are available upon reasonable request from the corresponding author.

## Abbreviations

The following abbreviations are used in this manuscript:

DSF	Double-screw fixation
SSBBC	Single-suture bone block cerclage
DSBBC	Double-suture bone block cerclage
